# Emerging roles of extracellular vesicles in cellular senescence and aging

**DOI:** 10.1111/acel.12734

**Published:** 2018-02-01

**Authors:** Masaki Takasugi

**Affiliations:** ^1^ Department of Biology University of Rochester Rochester NY USA

**Keywords:** aging, cellular senescence, cytoplasmic DNA, EphA2, extracellular vesicles, telomere

## Abstract

Cellular senescence is a cellular program that prevents the proliferation of cells at risk of neoplastic transformation. On the other hand, age‐related accumulation of senescent cells promotes aging at least partially due to the senescence‐associated secretory phenotype, whereby cells secrete high levels of inflammatory cytokines, chemokines, and matrix metalloproteinases. Emerging evidence, however, indicates that extracellular vesicles (EVs) are important mediators of the effects of senescent cells on their microenvironment. Senescent cells secrete more EphA2 and DNA via EVs, which can promote cancer cell proliferation and inflammation, respectively. Extracellular vesicles secreted from DNA‐damaged cells can also affect telomere regulation. Furthermore, it has now become clear that EVs actually play important roles in many aspects of aging. This review is intended to summarize these recent progresses, with emphasis on relationships between cellular senescence and EVs.

## 1 INTRODUCTION

### 1.1 Cellular senescence and senescence‐associated secretory phenotype (SASP)

Cellular senescence prevents the proliferation of cells exposed to potentially oncogenic stresses, such as DNA‐damaging reagents, irradiation, telomere shortening, and oncogene activation (Kuilman, Michaloglou, Mooi & Peeper, 2010; Rodier & Campisi, 2011). Mutations in genes essential for the senescence‐induced cell cycle arrest predispose cells to immortalization and shorten lifespan by increasing cancer incidence. However, cellular senescence not only arrests the cell cycle but also changes how the cell impacts its microenvironment. The way in which senescent cells influence their microenvironment is highly context dependent (Muñoz‐Espín & Serrano, 2014). It promotes tumor development in many cases, but can also be tumor suppressive in certain circumstances. Removal of senescent cells that accumulated in the body during aging alleviates atherosclerosis, hepatic steatosis, tumor development, and functional declines of heart, kidney, and fat tissues, resulting in prolonged healthspan and lifespan (Baar et al., 2017; Baker et al., 2016; Childs et al., 2016; Ogrodnik et al., 2017). These effects may be attributable to so‐called senescence‐associated secretory phenotype (SASP), whereby cells secrete high levels of inflammatory cytokines, chemokines, growth factors, and metalloproteinases (Coppé, Desprez, Krtolica & Campisi, 2010; Coppé et al., 2008). Among these SASP factors, for example, amphiregulin promotes cancer cell proliferation (Bavik et al., 2006); IL‐6, IL‐8, and CCL2 promote cancer cell invasion (Coppé et al., 2008; Ohanna et al., 2011); VEGF promotes angiogenesis (Coppé, Kauser, Campisi & Beauséjour, 2006); IL‐6, IL‐8, IGFBP7, and PAI‐1 reinforce cellular senescence (Acosta et al., 2008; Kortlever, Higgins & Bernards, 2006; Kuilman et al., 2008; Wajapeyee, Serra, Zhu, Mahalingam & Green, 2008); TGF‐β family ligands, CCL2, CCL20, and VEGF transmit cellular senescence (Acosta et al., 2013); and PDGF‐AA promotes wound healing (Demaria et al., 2014). In senescent cells, these SASP factors are mostly activated at transcriptional level by NF‐κB and C/EBPβ (Acosta et al., 2008; Chien et al., 2011; Kuilman et al., 2008).

### 1.2 Extracellular vesicles (EVs)

**Figure 1:**
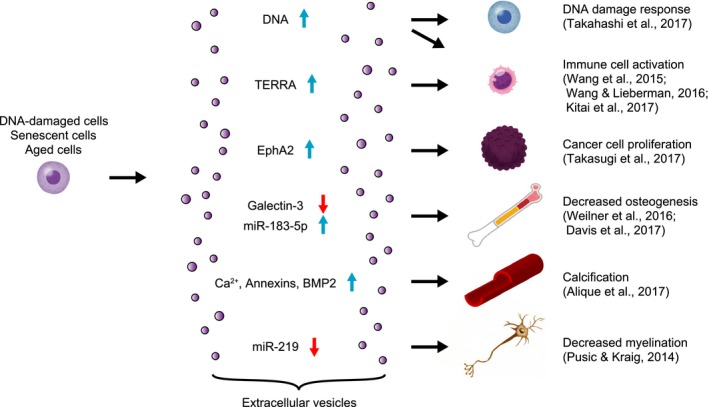
The functions of extracellular vesicles (EVs) secreted from DNA‐damaged, senescent, or aged cells

Although the involvement of typical secretory proteins in the non‐cell‐autonomous effects of senescent cells has been well studied, the functions of membrane‐enclosed vesicles secreted by senescent cells have not been studied until recently. These extracellular vesicles (EVs) were once thought to be cellular trash, but now it is clear that they are critical mediators in intercellular communication (Raposo & Stoorvogel, 2013). Emerging evidence indicates that EVs also play important roles in cellular senescence and aging (Figure 1). This field is rapidly advancing especially since Valadi et al. (2007) reported that EVs deliver functional RNA to the recipient cells. Extracellular vesicles contain a huge variety of proteins and nucleic acids in a cell type‐dependent manner. Extracellular vesicles are actually heterogeneous population (Bobrie, Colombo, Krumeich, Raposo & Théry, 2012; Kowal et al., 2016; Raposo & Stoorvogel, 2013) that can be classified based on their origin. The most well‐studied EVs are exosomes (Colombo, Raposo & Théry, 2014), which originate as intraluminal vesicles (ILVs) in the late endosomal compartment through inward budding of the endosomal membranes. Exosomes are secreted from the cells once the vesicle containing ILVs, namely multivesicular body (MVB), fuse with the plasma membrane. Biogenesis of ILVs involves ESCRT complexes (Colombo et al., 2013), tetraspanins (Andreu & Yáñez‐Mó, 2014), ceramide (Trajkovic et al., 2008), or their combination. In the ESCRT‐dependent pathway, ESCRT‐0 complex first recruits cargos and ESCRT‐I/‐II complexes to the endosomal membrane, then ESCRT‐I/‐II complexes induce bud formation, and ESCRT‐III drives scission of ILVs. Ubiquitinated proteins are recognized by the ESCRT‐0/‐I/‐II complexes during these processes and are enriched in exosomes (Hanson & Cashikar, 2012). In what way tetraspanins and ceramide induce ILV formation is not well understood. However, it has been shown that tetraspanin CD81 is required for sorting its interacting proteins into exosomes (Perez‐Hernandez et al., 2013). There are also some mechanisms that target specific miRNAs to exosomes. RNA‐binding proteins such as hnRNPA2B1, YBX1, SYNCRIP, and MEX3C are at least a few involved in this process (Lu et al., 2017; Santangelo et al., 2016; Shurtleff, Temoche‐Diaz, Karfilis, Ri & Schekman, 2016; Villarroya‐Beltri et al., 2013). The Rab family proteins that regulate intracellular vesicular trafficking required for exosome secretion include at least Rab7 (Baietti et al., 2012), Rab11 (Savina, Vidal & Colombo, 2002), Rab27A/B (Ostrowski et al., 2010), and Rab35 (Hsu et al., 2010). Based on their localization, it is proposed that Rab27A/B regulates the plasma membrane trafficking of more matured MVB compared to Rab11 and Rab35 (Kowal, Tkach & Théry, 2014). The exosome biogenesis pathway that predominates depends on the cell type. Once secreted, exosomes can be taken up by recipient cells through endocytosis, phagocytosis, macropinocytosis, or membrane fusion (Mulcahy, Pink & Carter, 2014). Exosomal membrane proteins have the same topology as the cells and can target exosomes to specific cell type. Other classes of EVs include, but are not limited to, microvesicles and apoptotic bodies. Although many proteins such as ESCRT proteins and tetraspanins are enriched in exosomes and are used as exosome markers, they are not necessarily strictly exosome specific (Witwer et al., 2013). It is therefore difficult to attribute the given function of EVs exclusively to specific EV subpopulation. For this reason, although most of the references cited herein are focused on exosomes or at least exosome‐like EVs, the general term “EVs” is used in this review instead of referring to specific EV subpopulations.

## 2 CELLULAR SENESCENCE INCREASES EV SECRETION

Senescence‐associated increase in EV secretion was first described by Lehmann et al. (2008). This increase seems to be a general feature of cellular senescence and has been observed in fibroblasts, epithelial cells, and cancer cells. Cellular senescence triggered by serial passaging, irradiation, DNA‐damaging reagents, and oncogenic Ras expression all enhance EV secretion, with an increase of more than 10‐fold in most cases (Lehmann et al., 2008; Takahashi et al., 2017; Takasugi et al., 2017). This increase is at least partially mediated by p53 (Lehmann et al., 2008) and one of its targets, TSAP6 (Lespagnol et al., 2008; Yu, Harris & Levine, 2006), although the mechanism whereby TSAP6 regulates EV secretion is not well understood. In addition, p53 induces several genes involved in endosome regulation, such as Caveolin‐1 and the ESCRT‐III subunit Chmp4 (Yu, Riley & Levine, 2009). It has also been reported that cellular senescence of cervical cancer cells induces expression of several Rab genes including Rab5B, which plays important roles in endosome maturation (Wells et al., 2003). Another report showed that expression of Rab27B, which promotes the secretion of EVs, is enhanced in senescent fibroblasts (Fujii et al., 2006). It is known that EV secretion contributes to the clearance of harmful molecules in the cells (Desdín‐Micó & Mittelbrunn, 2017), such as cytoplasmic DNA. As described later in this review, EV‐mediated removal of cytoplasmic DNA is essential for the survival of senescent cells (Takahashi et al., 2017), which may explain why EV secretion is increased in senescent cells.

## 3 SENESCENT CELLS STIMULATE MITOGENIC PATHWAY IN CANCER CELLS THROUGH EV‐ASSOCIATED EPHA2

A recent study has shown that EVs are important mediators of the protumorigenic effect of the senescent cell secretome (Takasugi et al., 2017). In this study, the authors first showed that conditioned media prepared from senescent cells lose their proproliferative effect on MCF‐7 breast cancer cells when EVs are removed from the conditioned media. Conversely, EVs purified from senescent cells promote the proliferation of several types of cancer cells. Proteomic profiling of senescent and nonsenescent cell EVs revealed drastic differences in their contents, including the enrichment of receptor tyrosine kinase EphA2 in senescent cell EVs. The binding of EphA2 to ephrin ligands can activate Erk pathway through so‐called reverse signals in ephrin‐expressing cells. Indeed, EphA2 was found to be responsible for the proproliferative effect of senescent cell EVs. Remarkably, an antibody targeted to the extracellular region of ephrin‐A1 significantly suppresses the proproliferative effect of conditioned medium of senescent cells regardless of whether cellular senescence was induced by serial passaging, oncogenic Ras, or doxorubicin. Interestingly, EphA2 tends to be upregulated in breast and ovary cancer stroma. It is also known that ephrin‐A1 is upregulated in many types of cancer cells (Beauchamp & Debinski, 2012). Moreover, a recent study showed that the plasma levels of EV‐associated EphA2 are increased in pancreatic cancer patients (Liang et al., 2017). These facts suggest the contribution of EV‐associated EphA2 to cancer development.

Selective increase of EphA2 in senescent cell EVs is regulated by both expression level and post‐translational modification of EphA2. It is known that activated p53 increases EphA2 expression (Dohn, Jiang & Chen, 2001). In some types of senescent cells, however, EphA2 is increased in EVs without any changes in its expression level, such as in Ras‐induced senescent cells. PTP1B phosphatase, which suppresses autophosphorylation of EphA2 (Sabet et al., 2015), is oxidatively inactivated in senescent cells due to elevated reactive oxygen species (ROS) levels, resulting in endosomal internalization of EphA2. Endosomal internalization is the first step to sort EphA2 into exosomes, and its inhibition through PTP1B overexpression abrogates EV‐mediated secretion of EphA2. Therefore, non‐cell‐autonomous protumorigenic effects of senescent cells are not only regulated by known mechanisms of SASP regulation, but also by ROS‐regulated cargo sorting into EVs.

## 4 EV‐MEDIATED SECRETION OF CYTOPLASMIC DNA: IMPLICATIONS IN INFLAMMATION

Extracellular vesicles contain various lengths of genomic DNA fragments without any noticeable sequence specificity (Kahlert et al., 2014; Thakur et al., 2014) and seem to be one of the major routes of DNA secretion (Fernando, Jiang, Krzyzanowski & Ryan, 2017). DNA associated with the outer membrane of EVs is larger and mostly double‐stranded, whereas both single‐stranded DNA and double‐stranded DNA are abundant inside the EVs (Thakur et al., 2014). Extracellular vesicle‐mediated DNA secretion increases upon cellular senescence (Takahashi et al., 2017). Intriguingly, γH2AX‐positive cytoplasmic chromatin fragments appear in senescent cells, suggesting that damaged DNA may be the major source of EV‐associated DNA in senescent cells (Ivanov et al., 2013). Apoptotic DNA fragments, however, are unlikely to be the major source of EV‐associated DNA (Takahashi et al., 2017). Inhibition of EV‐mediated DNA secretion induces apoptosis in senescent cells (Takahashi et al., 2017), although the autophagy–lysosomal pathway also removes cytosolic DNA (Ivanov et al., 2013; Lan, Londoño, Bouley, Rooney & Hacohen, 2014). Even in nonsenescent cells, inhibition of EV secretion increases cytosolic DNA and induces apoptosis and/or growth arrest in cGAS‐STING‐dependent manner (Takahashi et al., 2017). cGAS directly binds to cytosolic DNA and thereby activates and synthesizes cyclic GMP‐AMP (cGAMP) from ATP and GTP. Cyclic GMP‐AMP binds and induces a conformational change in the endoplasmic reticulum‐membrane adaptor STING. STING then activates transcription factors such as IRF3, NF‐κB, and STAT6 through TBK1 and IKK (Chen, Sun & Chen, 2016). Emerging studies revealed that STING is critical for the induction of SASP genes and establishment of cellular senescence in vitro and in vivo (Dou et al., 2017; Glück et al., 2017; Yang, Wang, Ren, Chen & Chen, 2017). Therefore, elevated cytosolic DNA is important for the senescent phenotype, while some of it has to be removed through EVs or autophagy–lysosomal pathway for cell survival. Cancer cells, on the other hand, are more tolerant to cytosolic DNA. It has been shown that inhibition of EV secretion does not affect proliferation of HEK293T, HeLa, and U2OS cells (Takahashi et al., 2017). Nonetheless, inflammatory gene expressions in breast, pancreatic, prostate cancers, and melanoma correlate with STING expression (Dou et al., 2017). Its impact on prognosis, however, depends largely on the type of cancer (Yang et al., 2017). The function of EV‐associated DNA is also context dependent. In hormone receptor‐positive breast cancer cells, cell‐free DNA including EV‐associated DNA promotes cell proliferation (Wang et al., 2017). On the other hand, it has been suggested that EV‐associated DNA activates antitumor immunity. Antitumor agent topotecan (TPT), an inhibitor of topoisomerase I, induces EV‐associated DNA secretion in cancer cells, which in turn activates dendritic cells in a STING‐dependent manner. Notably, TPT requires STING to activate immune cells and inhibit tumor growth in mice bearing E0771 breast cancer cells (Kitai et al., 2017). It has also been reported that topoisomerase I inhibitor CPT11 induces EV‐associated DNA secretion from cells proliferating in the intestine, which then activates immune cells and causes diarrhea (Lian et al., 2017). In this case, another DNA sensor, AIM2, primarily mediates the effects of EV‐associated DNA. In spite of these findings, how EV‐associated DNA secreted from senescent cells impacts their microenvironment is still largely unknown. It has been suggested that EV‐associated DNAs secreted from senescent cells trigger a DNA damage response in recipient cells (Takahashi et al., 2017), and therefore, it may contribute to reinforce or transmit cellular senescence.

## 5 EVS SECRETED FROM DNA‐DAMAGED CELLS INDUCE TELOMERE DYSREGULATION

Emerging evidence suggests the involvement of EVs in telomere regulation. It has been shown that EVs secreted from irradiated breast cancer cells suppress telomerase activity and induce telomere shortening in recipient cells (Al‐Mayah et al., 2017). Both RNA and protein contents of EVs seem to be contributing to this effect. Another recent paper showed the enrichment of telomeric repeat‐containing RNA (TERRA) in EVs (Wang & Lieberman, 2016; Wang et al., 2015). In cells, TERRA is known to have diverse functions in telomere regulation such as repression of telomerase activity (Azzalin & Lingner, 2015). Impairment of telomere integrity by TRF2 inhibition results in increased TERRA levels in cells and EVs (Wang & Lieberman, 2016). Although it is still unclear whether EV‐mediated TERRA secretion is enhanced in the context of cellular senescence or aging, it has been shown that telomere shortening, which is involved in replicative senescence and aging, increases expression level of TERRA (Cusanelli, Romero & Chartrand, 2013). Extracellular vesicle‐associated TERRA was found to induce inflammatory gene expressions in recipient cells (Wang & Lieberman, 2016; Wang et al., 2015). However, EV‐associated TERRA is significantly shorter than intracellular TERRA, and thus, whether it also affects telomere regulation is not clear.

## 6 AGE‐RELATED CHANGES IN EVS

A cross‐sectional and longitudinal study by Eitan et al. (2017) showed that plasma EV concentration decreases with human age, at least from early 30s to late 60s. They showed that monocyte and B cells but not T cells internalize plasma EVs and that B cells from the elderly uptake more EVs. Interestingly, smoking and higher BMI are both positively correlated with plasma EV concentration. Considering that smoking and obesity trigger cellular senescence in specific tissues (Tsuji, Aoshiba & Nagai, 2004; Yoshimoto et al., 2013), this finding may suggest that EVs secreted from accumulated senescent cells can account for substantial portions of circulating EVs. In addition to the concentration, aging alters RNA and protein composition of EVs. Galectin‐3, which plays a role in osteoblast maturation, is reduced in the plasma EVs of elderly people. Cellular senescence decreases EV‐mediated galectin‐3 secretion of endothelial cells and may be responsible for its age‐related reduction. Plasma EVs isolated from young but not elderly donors promote the osteogenic differentiation of mesenchymal stem cells in a galectin‐3‐dependent manner (Weilner et al., 2016). These findings suggest that reduced EV‐mediated secretion of galectin‐3 is one of the causes of the age‐related decline in bone formation. Another paper showed that large EVs isolated from elderly subjects’ plasma or senescent endothelial cells promote the calcification of human aortic smooth muscle cells (Alique et al., 2017). These large EVs secreted from senescent endothelial cells contain increased amounts of calcium, calcium‐binding annexins, and BMP2, all of which are involved in vascular calcification. In addition to the plasma EVs, bone marrow EVs of the elderly also show anti‐osteogenic effect. Extracellular vesicles purified from the elderly suppress cell proliferation and osteogenic differentiation of bone marrow stromal cells (Davis et al., 2017). The amount of miR‐183‐5p is increased in aged bone marrow EVs, and the overexpression of miR‐183‐5p in bone marrow stromal cells mimics the effects of aged bone marrow EVs. This increase in EV‐associated miR‐183‐5p is at least partially attributable to enhanced ROS levels in the aged bone marrow (Davis et al., 2017). Consistently, it has been shown that ROS‐induced senescence enhances miR‐183 expression (Li, Luna, Qiu, Epstein & Gonzalez, 2009). EV‐associated miRNAs‐associated miRNAs are also involved in brain aging. Serum EVs isolated from young rats promote oligodendrocyte precursor cell (OPC) differentiation and remyelination in slice cultures. Moreover, nasal administration of young EVs increases myelination in aged rat brain. Mechanistically, it has been shown that young EVs contain high levels of miR‐219, which reduces the expression of inhibitory regulators of OPC differentiation. Indeed, transfection of miR‐219 inhibitor along with the administration of young EVs abolishes the beneficial effects of EVs (Pusic & Kraig, 2014). Finally, a recent report by Zhang et al. (2017) demonstrated that administration of EVs isolated from young cells ameliorates age‐related functional declines in mice. This study focused on hypothalamic neural stem cells (htNSCs) and showed that the number of htNSC and thus htNSC‐derived EVs decrease with age. Selective ablation of htNSCs accelerates age‐related functional declines such as decreases in treadmill performance and cognitive functions, resulting in a shorter lifespan. Administration of EVs isolated from newborn‐derived htNSCs suppresses this acceleration of aging to at least some extent. The number and miRNA content of htNSC‐derived EVs are much higher than those of astrocyte‐derived EVs. Indeed, htNSC‐derived EVs significantly contribute to the miRNA profile in the cerebrospinal fluid, suggesting that miRNA may be mediating the anti‐aging effects of htNSC‐derived EVs. As summarized above, EVs have been implicated in many aspects of aging in the past few years. At least some of these age‐related changes in EVs could be attributed to cellular senescence, further supporting the usefulness of targeting senescent cells as a treatment for aging and age‐related diseases.

## 7 CONCLUSION

The importance of cellular senescence in aging and age‐related diseases is increasingly appreciated, and methods to remove senescent cells or suppress SASP are currently under intensive studies. A better understanding of how senescent cells impact their microenvironment is necessary for efficient drug development. Notably, recent findings implicate senescent cell EVs in cancer development (Takasugi et al., 2017), vascular calcification (Alique et al., 2017), and age‐related decline in bone formation (Weilner et al., 2016). Increased secretion of EV‐associated DNA from senescent cells (Takahashi et al., 2017) is likely to be pro‐inflammatory (Kitai et al., 2017; Lian et al., 2017) and may contribute to age‐related chronic inflammation. Whether senescent cell EVs promote or suppress cancer development may be context dependent. Extracellular vesicle‐associated EphA2 secreted from senescent cells stimulates mitogenic pathway in cancer cells (Takasugi et al., 2017). Extracellular vesicle‐associated DNA can also promote cancer cell proliferation through unknown mechanisms (Wang et al., 2017). On the other hand, EV‐associated DNA can induce antitumor immunity (Kitai et al., 2017). Despite these progresses, it should be noted that the functions of senescent cell EVs are still understudied, at least partially due to inadequate understanding of EVs themselves. This research field is immature and the methods used are not sufficiently standardized yet. In this regard, guidelines provided by the International Society for Extracellular Vesicles would be useful for researchers (Mateescu et al., 2017; Witwer et al., 2013). Nevertheless, EVs have now shown to be critical players in cellular senescence and aging, and more functions will be revealed in the future as the EV research field matures. As there seem to be substantial differences between regulation of EVs and known SASP factors in senescent cells, further investigation of EVs will enable more specific modulation of the senescent cell secretome.

## CONFLICT OF INTEREST

None declared.

## References

[acel12734-bib-0001] Acosta, J. C. , Banito, A. , Wuestefeld, T. , Georgilis, A. , Janich, P. , Morton, J. P. , … Gil, J. (2013). A complex secretory program orchestrated by the inflammasome controls paracrine senescence. Nature Cell Biology, 15, 978–990. https://doi.org/10.1038/ncb2784 23770676 10.1038/ncb2784 PMC3732483

[acel12734-bib-0002] Acosta, J. C. , O'Loghlen, A. , Banito, A. , Guijarro, M. V. , Augert, A. , Raguz, S. , … Gil, J. (2008). Chemokine signaling via the CXCR2 receptor reinforces senescence. Cell, 133, 1006–1018. https://doi.org/10.1016/j.cell.2008.03.038 18555777 10.1016/j.cell.2008.03.038

[acel12734-bib-0003] Alique, M. , Ruíz‐Torres, M. P. , Bodega, G. , Noci, M. V. , Troyano, N. , Bohórquez, L. , … Ramírez, R. (2017). Microvesicles from the plasma of elderly subjects and from senescent endothelial cells promote vascular calcification. Aging (Albany NY), 9, 778–789. 28278131 10.18632/aging.101191 PMC5391231

[acel12734-bib-0004] Al‐Mayah, A. H. J. , Bright, S. J. , Bowler, D. A. , Slijepcevic, P. , Goodwin, E. , & Kadhim, M. A. (2017). Exosome‐mediated telomere instability in human breast epithelial cancer cells after X irradiation. Radiation Research, 187, 98–106. https://doi.org/10.1667/RR14201.1 27959588 10.1667/RR14201.1

[acel12734-bib-0005] Andreu, Z. , & Yáñez‐Mó, M. (2014). Tetraspanins in extracellular vesicle formation and function. Frontiers in Immunology, 5, 442. 25278937 10.3389/fimmu.2014.00442 PMC4165315

[acel12734-bib-0006] Azzalin, C. M. , & Lingner, J. (2015). Telomere functions grounding on TERRA firma. Trends in Cell Biology, 25, 29–36. https://doi.org/10.1016/j.tcb.2014.08.007 25257515 10.1016/j.tcb.2014.08.007

[acel12734-bib-0007] Baar M. P. , Brandt R. M. C. , Putavet D. A. , Klein J. D. D. , Derks K. W. J. , Bourgeois B. R. M. , … de Keizer P. L. J. (2017). Targeted apoptosis of senescent cells restores tissue homeostasis in response to chemotoxicity and aging. Cell 169, 132–147.e16. https://doi.org/10.1016/j.cell.2017.02.031 28340339 10.1016/j.cell.2017.02.031 PMC5556182

[acel12734-bib-0008] Baietti, M. F. , Zhang, Z. , Mortier, E. , Melchior, A. , Degeest, G. , Geeraerts, A. , … David, G. (2012). Syndecan‐syntenin‐ALIX regulates the biogenesis of exosomes. Nature Cell Biology, 14, 677–685. https://doi.org/10.1038/ncb2502 22660413 10.1038/ncb2502

[acel12734-bib-0009] Baker, D. J. , Childs, B. G. , Durik, M. , Wijers, M. E. , Sieben, C. J. , Zhong, J. , … van Deursen, J. M. (2016). Naturally occurring p16(Ink4a)‐positive cells shorten healthy lifespan. Nature, 530, 184–189. https://doi.org/10.1038/nature16932 26840489 10.1038/nature16932 PMC4845101

[acel12734-bib-0010] Bavik, C. , Coleman, I. , Dean, J. P. , Knudsen, B. , Plymate, S. , & Nelson, P. S. (2006). The gene expression program of prostate fibroblast senescence modulates neoplastic epithelial cell proliferation through paracrine mechanisms. Cancer Research, 66, 794–802. https://doi.org/10.1158/0008-5472.CAN-05-1716 16424011 10.1158/0008-5472.CAN-05-1716

[acel12734-bib-0011] Beauchamp, A. , & Debinski, W. (2012). Ephs and ephrins in cancer: ephrin‐A1 signalling. Seminars in Cell & Developmental Biology, 23, 109–115. https://doi.org/10.1016/j.semcdb.2011.10.019 22040911 10.1016/j.semcdb.2011.10.019 PMC3288643

[acel12734-bib-0012] Bobrie A. , Colombo M. , Krumeich S. , Raposo G. , & Théry C. (2012). Diverse subpopulations of vesicles secreted by different intracellular mechanisms are present in exosome preparations obtained by differential ultracentrifugation. Journal of Extracellular Vesicles 1, 18397 https://doi.org/10.3402/jev.v1i0.18397 10.3402/jev.v1i0.18397 PMC3760636 24009879

[acel12734-bib-0013] Chen, Q. , Sun, L. , & Chen, Z. J. (2016). Regulation and function of the cGAS‐STING pathway of cytosolic DNA sensing. Nature Immunology, 17, 1142–1149. https://doi.org/10.1038/ni.3558 27648547 10.1038/ni.3558

[acel12734-bib-0014] Chien, Y. , Scuoppo, C. , Wang, X. , Fang, X. , Balgley, B. , Bolden, J. E. , … Lowe, S. W. (2011). Control of the senescence‐associated secretory phenotype by NF‐κB promotes senescence and enhances chemosensitivity. Genes & Development, 25, 2125–2136. https://doi.org/10.1101/gad.17276711 21979375 10.1101/gad.17276711 PMC3205583

[acel12734-bib-0015] Childs, B. G. , Baker, D. J. , Wijshake, T. , Conover, C. A. , Campisi, J. , & van Deursen, J. M. (2016). Senescent intimal foam cells are deleterious at all stages of atherosclerosis. Science, 354, 472–477. https://doi.org/10.1126/science.aaf6659 27789842 10.1126/science.aaf6659 PMC5112585

[acel12734-bib-0016] Colombo, M. , Moita, C. , van Niel, G. , Kowal, J. , Vigneron, J. , Benaroch, P. , … Raposo, G. (2013). Analysis of ESCRT functions in exosome biogenesis, composition and secretion highlights the heterogeneity of extracellular vesicles. Journal of Cell Science, 126, 5553–5565. https://doi.org/10.1242/jcs.128868 24105262 10.1242/jcs.128868

[acel12734-bib-0017] Colombo, M. , Raposo, G. , & Théry, C. (2014). Biogenesis, secretion, and intercellular interactions of exosomes and other extracellular vesicles. Annual Review of Cell and Developmental Biology, 30, 255–289. https://doi.org/10.1146/annurev-cellbio-101512-122326 10.1146/annurev-cellbio-101512-122326 25288114

[acel12734-bib-0018] Coppé, J.‐P. , Desprez, P.‐Y. , Krtolica, A. , & Campisi, J. (2010). The senescence‐associated secretory phenotype: The dark side of tumor suppression. Annual Review of Pathology: Mechanisms of Disease, 5, 99–118. https://doi.org/10.1146/annurev-pathol-121808-102144 10.1146/annurev-pathol-121808-102144 PMC4166495 20078217

[acel12734-bib-0019] Coppé, J.‐P. , Kauser, K. , Campisi, J. , & Beauséjour, C. M. (2006). Secretion of vascular endothelial growth factor by primary human fibroblasts at senescence. Journal of Biological Chemistry, 281, 29568–29574. https://doi.org/10.1074/jbc.M603307200 16880208 10.1074/jbc.M603307200

[acel12734-bib-0020] Coppé, J.‐P. , Patil, C. K. , Rodier, F. , Sun, Y. , Muñoz, D. P. , Goldstein, J. , … Campisi, J. (2008). Senescence‐associated secretory phenotypes reveal cell‐nonautonomous functions of oncogenic RAS and the p53 tumor suppressor. PLoS Biology, 6, 2853–2868. 19053174 10.1371/journal.pbio.0060301 PMC2592359

[acel12734-bib-0021] Cusanelli, E. , Romero, C. A. P. , & Chartrand, P. (2013). Telomeric noncoding RNA TERRA is induced by telomere shortening to nucleate telomerase molecules at short telomeres. Molecular Cell, 51, 780–791. https://doi.org/10.1016/j.molcel.2013.08.029 24074956 10.1016/j.molcel.2013.08.029

[acel12734-bib-0022] Davis C. , Dukes A. , Drewry M. , Helwa I. , Johnson M. H. , Isales C. M. , … Hamrick M. W. (2017). MicroRNA‐183‐5p increases with age in bone‐derived extracellular vesicles, suppresses bone marrow stromal (stem) cell proliferation, and induces stem cell senescence. Tissue Engineering Part A 23, 1231–1240. https://doi.org/10.1089/ten.tea.2016.0525 28363268 10.1089/ten.tea.2016.0525 PMC5689127

[acel12734-bib-0023] Demaria, M. , Ohtani, N. , Youssef, S. A. , Rodier, F. , Toussaint, W. , Mitchell, J. R. , … Campisi, J. (2014). An essential role for senescent cells in optimal wound healing through secretion of PDGF‐AA. Developmental Cell, 31, 722–733. https://doi.org/10.1016/j.devcel.2014.11.012 25499914 10.1016/j.devcel.2014.11.012 PMC4349629

[acel12734-bib-0024] Desdín‐Micó, G. , & Mittelbrunn, M. (2017). Role of exosomes in the protection of cellular homeostasis. Cell Adhesion and Migration, 11, 127–134. https://doi.org/10.1080/19336918.2016.1251000 27875097 10.1080/19336918.2016.1251000 PMC5351736

[acel12734-bib-0025] Dohn, M. , Jiang, J. , & Chen, X. (2001). Receptor tyrosine kinase EphA2 is regulated by p53‐family proteins and induces apoptosis. Oncogene, 20, 6503–6515. https://doi.org/10.1038/sj.onc.1204816 11641774 10.1038/sj.onc.1204816

[acel12734-bib-0026] Dou, Z. , Ghosh, K. , Vizioli, M. G. , Zhu, J. , Sen, P. , Wangensteen, K. J. , … Berger, S. L. (2017). Cytoplasmic chromatin triggers inflammation in senescence and cancer. Nature, 550, 402–406. https://doi.org/10.1038/nature24050 28976970 10.1038/nature24050 PMC5850938

[acel12734-bib-0027] Eitan, E. , Green, J. , Bodogai, M. , Mode, N. A. , Bæk, R. , Jørgensen, M. M. , … Evans, M. K. (2017). Age‐related changes in plasma extracellular vesicle characteristics and internalization by leukocytes. Scientific Reports, 7, 1342 https://doi.org/10.1038/s41598-017-01386-z 28465537 10.1038/s41598-017-01386-z PMC5430958

[acel12734-bib-0028] Fernando, M. R. , Jiang, C. , Krzyzanowski, G. D. , & Ryan, W. L. (2017). New evidence that a large proportion of human blood plasma cell‐free DNA is localized in exosomes. PLoS One, 12, e0183915 https://doi.org/10.1371/journal.pone.0183915 28850588 10.1371/journal.pone.0183915 PMC5574584

[acel12734-bib-0029] Fujii, M. , Kawai, Y. , Endoh, M. , Hossain, M. N. , Nakabayashi, K. , & Ayusawa, D. (2006). Expression of RAB27B is up‐regulated in senescent human cells. Mechanisms of Ageing and Development, 127, 639–642. https://doi.org/10.1016/j.mad.2006.03.001 16620919 10.1016/j.mad.2006.03.001

[acel12734-bib-0030] Glück, S. , Guey, B. , Gulen, M. F. , Wolter, K. , Kang, T.‐W. , Schmacke, N. A. , … Ablasser, A. (2017). Innate immune sensing of cytosolic chromatin fragments through cGAS promotes senescence. Nature Cell Biology, 19, 1061–1070. https://doi.org/10.1038/ncb3586 28759028 10.1038/ncb3586 PMC5826565

[acel12734-bib-0031] Hanson, P. I. , & Cashikar, A. (2012). Multivesicular body morphogenesis. Annual Review of Cell and Developmental Biology, 28, 337–362. https://doi.org/10.1146/annurev-cellbio-092910-154152 10.1146/annurev-cellbio-092910-154152 22831642

[acel12734-bib-0032] Hsu, C. , Morohashi, Y. , Yoshimura, S.‐I. , Manrique‐Hoyos, N. , Jung, S. , Lauterbach, M. A. , … Simons, M. (2010). Regulation of exosome secretion by Rab35 and its GTPase‐activating proteins TBC1D10A‐C. Journal of Cell Biology, 189, 223–232. https://doi.org/10.1083/jcb.200911018 20404108 10.1083/jcb.200911018 PMC2856897

[acel12734-bib-0033] Ivanov, A. , Pawlikowski, J. , Manoharan, I. , van Tuyn, J. , Nelson, D. M. , Rai, T. S. , … Adams, P. D. (2013). Lysosome‐mediated processing of chromatin in senescence. Journal of Cell Biology, 202, 129–143. https://doi.org/10.1083/jcb.201212110 23816621 10.1083/jcb.201212110 PMC3704985

[acel12734-bib-0034] Kahlert, C. , Melo, S. A. , Protopopov, A. , Tang, J. , Seth, S. , Koch, M. , … Kalluri, R. (2014). Identification of double‐stranded genomic DNA spanning all chromosomes with mutated KRAS and p53 DNA in the serum exosomes of patients with pancreatic cancer. Journal of Biological Chemistry, 289, 3869–3875. https://doi.org/10.1074/jbc.C113.532267 24398677 10.1074/jbc.C113.532267 PMC3924256

[acel12734-bib-0035] Kitai, Y. , Kawasaki, T. , Sueyoshi, T. , Kobiyama, K. , Ishii, K. J. , Zou, J. , … Kawai, T. (2017). DNA‐containing exosomes derived from cancer cells treated with topotecan activate a STING‐dependent pathway and reinforce antitumor immunity. Journal of Immunology, 198, 1649–1659. https://doi.org/10.4049/jimmunol.1601694 10.4049/jimmunol.1601694 28069806

[acel12734-bib-0036] Kortlever, R. M. , Higgins, P. J. , & Bernards, R. (2006). Plasminogen activator inhibitor‐1 is a critical downstream target of p53 in the induction of replicative senescence. Nature Cell Biology, 8, 877–884. https://doi.org/10.1038/ncb1448 16862142 10.1038/ncb1448 PMC2954492

[acel12734-bib-0037] Kowal, J. , Arras, G. , Colombo, M. , Jouve, M. , Morath, J. P. , Primdal‐Bengtson, B. , … Théry, C. (2016). Proteomic comparison defines novel markers to characterize heterogeneous populations of extracellular vesicle subtypes. Proceedings of the National Academy of Sciences of the United States of America, 113, E968–E977. https://doi.org/10.1073/pnas.1521230113 26858453 10.1073/pnas.1521230113 PMC4776515

[acel12734-bib-0038] Kowal, J. , Tkach, M. , & Théry, C. (2014). Biogenesis and secretion of exosomes. Current Opinion in Cell Biology, 29, 116–125. https://doi.org/10.1016/j.ceb.2014.05.004 24959705 10.1016/j.ceb.2014.05.004

[acel12734-bib-0039] Kuilman, T. , Michaloglou, C. , Mooi, W. J. , & Peeper, D. S. (2010). The essence of senescence. Genes & Development, 24, 2463–2479. https://doi.org/10.1101/gad.1971610 21078816 10.1101/gad.1971610 PMC2975923

[acel12734-bib-0040] Kuilman, T. , Michaloglou, C. , Vredeveld, L. C. W. , Douma, S. , van Doorn, R. , Desmet, C. J. , … Peeper, D. S. (2008). Oncogene‐induced senescence relayed by an interleukin‐dependent inflammatory network. Cell, 133, 1019–1031. https://doi.org/10.1016/j.cell.2008.03.039 18555778 10.1016/j.cell.2008.03.039

[acel12734-bib-0041] Lan, Y. Y. , Londoño, D. , Bouley, R. , Rooney, M. S. , & Hacohen, N. (2014). Dnase2a deficiency uncovers lysosomal clearance of damaged nuclear DNA via autophagy. Cell Reports, 9, 180–192. https://doi.org/10.1016/j.celrep.2014.08.074 25284779 10.1016/j.celrep.2014.08.074 PMC4555847

[acel12734-bib-0042] Lehmann, B. D. , Paine, M. S. , Brooks, A. M. , McCubrey, J. A. , Renegar, R. H. , Wang, R. , & Terrian, D. M. (2008). Senescence‐associated exosome release from human prostate cancer cells. Cancer Research, 68, 7864–7871. https://doi.org/10.1158/0008-5472.CAN-07-6538 18829542 10.1158/0008-5472.CAN-07-6538 PMC3845029

[acel12734-bib-0043] Lespagnol, A. , Duflaut, D. , Beekman, C. , Blanc, L. , Fiucci, G. , Marine, J.‐C. , … Telerman, A. (2008). Exosome secretion, including the DNA damage‐induced p53‐dependent secretory pathway, is severely compromised in TSAP6/Steap3‐null mice. Cell Death and Differentiation, 15, 1723–1733. https://doi.org/10.1038/cdd.2008.104 18617898 10.1038/cdd.2008.104

[acel12734-bib-0044] Li, G. , Luna, C. , Qiu, J. , Epstein, D. L. , & Gonzalez, P. (2009). Alterations in microRNA expression in stress‐induced cellular senescence. Mechanisms of Ageing and Development, 130, 731–741. https://doi.org/10.1016/j.mad.2009.09.002 19782699 10.1016/j.mad.2009.09.002 PMC2795064

[acel12734-bib-0045] Lian, Q. , Xu, J. , Yan, S. , Huang, M. , Ding, H. , Sun, X. , … Geng, M. (2017). Chemotherapy‐induced intestinal inflammatory responses are mediated by exosome secretion of double‐strand DNA via AIM2 inflammasome activation. Cell Research, 27, 784–800. https://doi.org/10.1038/cr.2017.54 28409562 10.1038/cr.2017.54 PMC5518874

[acel12734-bib-0046] Liang K. , Liu F. , Fan J. , Sun D. , Liu C. , Lyon C. J. , … Hu Y. (2017) Nanoplasmonic quantification of tumor‐derived extracellular vesicles in plasma microsamples for diagnosis and treatment monitoring. Nature Biomedical Engineering 1, 0021 https://doi.org/10.1038/s41551-016-0021 10.1038/s41551-016-0021 PMC5543996 28791195

[acel12734-bib-0047] Lu, P. , Li, H. , Li, N. , Singh, R. N. , Bishop, C. E. , Chen, X. , & Lu, B. (2017). MEX3C interacts with adaptor‐related protein complex 2 and involves in miR‐451a exosomal sorting. PLoS One, 12, e0185992 https://doi.org/10.1371/journal.pone.0185992 28982131 10.1371/journal.pone.0185992 PMC5628917

[acel12734-bib-0048] Mateescu B. , Kowal E. J. K. , van Balkom B. W. M. , Bartel S. , Bhattacharyya S. N. , Buzás E. I. , … Nolte‐’t Hoen E. N. M. (2017). Obstacles and opportunities in the functional analysis of extracellular vesicle RNA – an ISEV position paper. Journal of Extracellular Vesicles 6, 1286095 https://doi.org/10.1080/20013078.2017.1286095 28326170 10.1080/20013078.2017.1286095 PMC5345583

[acel12734-bib-0049] Mulcahy L. A. , Pink R. C. , & Carter D. R. F. (2014). Routes and mechanisms of extracellular vesicle uptake. Journal of Extracellular Vesicles 3, 24641 https://doi.org/10.3402/jev.v3.24641 10.3402/jev.v3.24641 PMC4122821 25143819

[acel12734-bib-0050] Muñoz‐Espín, D. , & Serrano, M. (2014). Cellular senescence: From physiology to pathology. Nature Reviews Molecular Cell Biology, 15, 482–496. https://doi.org/10.1038/nrm3823 24954210 10.1038/nrm3823

[acel12734-bib-0051] Ogrodnik, M. , Miwa, S. , Tchkonia, T. , Tiniakos, D. , Wilson, C. L. , Lahat, A. , … Jurk, D. (2017). Cellular senescence drives age‐dependent hepatic steatosis. Nature Communications, 8, 15691 https://doi.org/10.1038/ncomms15691 10.1038/ncomms15691 PMC5474745 28608850

[acel12734-bib-0052] Ohanna, M. , Giuliano, S. , Bonet, C. , Imbert, V. , Hofman, V. , Zangari, J. , … Bertolotto, C. (2011). Senescent cells develop a PARP‐1 and nuclear factor‐{kappa}B‐associated secretome (PNAS). Genes & Development, 25, 1245–1261. https://doi.org/10.1101/gad.625811 21646373 10.1101/gad.625811 PMC3127427

[acel12734-bib-0053] Ostrowski M. , Carmo N. B. , Krumeich S. , Fanget I. , Raposo G. , Savina A. , … Thery C. (2010). Rab27a and Rab27b control different steps of the exosome secretion pathway. Nature Cell Biology 12, 19–30; sup. pp 1–13. https://doi.org/10.1038/ncb2000 19966785 10.1038/ncb2000

[acel12734-bib-0054] Perez‐Hernandez, D. , Gutiérrez‐Vázquez, C. , Jorge, I. , López‐Martín, S. , Ursa, A. , Sánchez‐Madrid, F. , … Yáñez‐Mó, M. (2013). The intracellular interactome of tetraspanin‐enriched microdomains reveals their function as sorting machineries toward exosomes. Journal of Biological Chemistry, 288, 11649–11661. https://doi.org/10.1074/jbc.M112.445304 23463506 10.1074/jbc.M112.445304 PMC3636856

[acel12734-bib-0055] Pusic, A. D. , & Kraig, R. P. (2014). Youth and environmental enrichment generate serum exosomes containing miR‐219 that promote CNS myelination. Glia, 62, 284–299. https://doi.org/10.1002/glia.22606 24339157 10.1002/glia.22606 PMC4096126

[acel12734-bib-0056] Raposo, G. , & Stoorvogel, W. (2013). Extracellular vesicles: Exosomes, microvesicles, and friends. Journal of Cell Biology, 200, 373–383. https://doi.org/10.1083/jcb.201211138 23420871 10.1083/jcb.201211138 PMC3575529

[acel12734-bib-0057] Rodier, F. , & Campisi, J. (2011). Four faces of cellular senescence. Journal of Cell Biology, 192, 547–556. https://doi.org/10.1083/jcb.201009094 21321098 10.1083/jcb.201009094 PMC3044123

[acel12734-bib-0058] Sabet, O. , Stockert, R. , Xouri, G. , Brüggemann, Y. , Stanoev, A. , & Bastiaens, P. I. H. (2015). Ubiquitination switches EphA2 vesicular traffic from a continuous safeguard to a finite signalling mode. Nature Communications, 6, 8047 https://doi.org/10.1038/ncomms9047 10.1038/ncomms9047 PMC4560775 26292967

[acel12734-bib-0059] Santangelo, L. , Giurato, G. , Cicchini, C. , Montaldo, C. , Mancone, C. , Tarallo, R. , … Tripodi, M. (2016). The RNA‐binding protein SYNCRIP is a component of the hepatocyte exosomal machinery controlling microRNA sorting. Cell Reports, 17, 799–808. https://doi.org/10.1016/j.celrep.2016.09.031 27732855 10.1016/j.celrep.2016.09.031

[acel12734-bib-0060] Savina, A. , Vidal, M. , & Colombo, M. I. (2002). The exosome pathway in K562 cells is regulated by Rab11. Journal of Cell Science, 115, 2505–2515. 12045221 10.1242/jcs.115.12.2505

[acel12734-bib-0061] Shurtleff M. J. , Temoche‐Diaz M. M. , Karfilis K. V. , Ri S. , & Schekman R. (2016). Y‐box protein 1 is required to sort microRNAs into exosomes in cells and in a cell‐free reaction. eLife 5, e19276. 27559612 10.7554/eLife.19276 PMC5047747

[acel12734-bib-0062] Takahashi, A. , Okada, R. , Nagao, K. , Kawamata, Y. , Hanyu, A. , Yoshimoto, S. , … Hara, E. (2017). Exosomes maintain cellular homeostasis by excreting harmful DNA from cells. Nature Communications, 8, 15287 https://doi.org/10.1038/ncomms15287 10.1038/ncomms15287 PMC5440838 28508895

[acel12734-bib-0063] Takasugi, M. , Okada, R. , Takahashi, A. , Virya Chen, D. , Watanabe, S. , & Hara, E. (2017). Small extracellular vesicles secreted from senescent cells promote cancer cell proliferation through EphA2. Nature Communications, 8, 15729 https://doi.org/10.1038/ncomms15728 10.1038/ncomms15728 PMC5467215 28585531

[acel12734-bib-0064] Thakur, B. K. , Zhang, H. , Becker, A. , Matei, I. , Huang, Y. , Costa‐Silva, B. , … Lyden, D. (2014). Double‐stranded DNA in exosomes: A novel biomarker in cancer detection. Cell Research, 24, 766–769. https://doi.org/10.1038/cr.2014.44 24710597 10.1038/cr.2014.44 PMC4042169

[acel12734-bib-0065] Trajkovic, K. , Hsu, C. , Chiantia, S. , Rajendran, L. , Wenzel, D. , Wieland, F. , … Simons, M. (2008). Ceramide triggers budding of exosome vesicles into multivesicular endosomes. Science, 319, 1244–1247. https://doi.org/10.1126/science.1153124 18309083 10.1126/science.1153124

[acel12734-bib-0066] Tsuji, T. , Aoshiba, K. , & Nagai, A. (2004). Cigarette smoke induces senescence in alveolar epithelial cells. American Journal of Respiratory Cell and Molecular Biology, 31, 643–649. https://doi.org/10.1165/rcmb.2003-0290OC 15333326 10.1165/rcmb.2003-0290OC

[acel12734-bib-0067] Valadi, H. , Ekström, K. , Bossios, A. , Sjöstrand, M. , Lee, J. J. , & Lötvall, J. O. (2007). Exosome‐mediated transfer of mRNAs and microRNAs is a novel mechanism of genetic exchange between cells. Nature Cell Biology, 9, 654–659. https://doi.org/10.1038/ncb1596 17486113 10.1038/ncb1596

[acel12734-bib-0068] Villarroya‐Beltri, C. , Gutiérrez‐Vázquez, C. , Sánchez‐Cabo, F. , Pérez‐Hernández, D. , Vázquez, J. , Martin‐Cofreces, N. , … Sánchez‐Madrid, F. (2013). Sumoylated hnRNPA2B1 controls the sorting of miRNAs into exosomes through binding to specific motifs. Nature Communications, 4, 2980. 10.1038/ncomms3980 PMC3905700 24356509

[acel12734-bib-0069] Wajapeyee, N. , Serra, R. W. , Zhu, X. , Mahalingam, M. , & Green, M. R. (2008). Oncogenic BRAF induces senescence and apoptosis through pathways mediated by the secreted protein IGFBP7. Cell, 132, 363–374. https://doi.org/10.1016/j.cell.2007.12.032 18267069 10.1016/j.cell.2007.12.032 PMC2266096

[acel12734-bib-0070] Wang, Z. , Deng, Z. , Dahmane, N. , Tsai, K. , Wang, P. , Williams, D. R. , … Lieberman, P. M. (2015). Telomeric repeat‐containing RNA (TERRA) constitutes a nucleoprotein component of extracellular inflammatory exosomes. Proceedings of the National Academy of Sciences of the United States of America, 112, E6293–E6300. https://doi.org/10.1073/pnas.1505962112 26578789 10.1073/pnas.1505962112 PMC4655533

[acel12734-bib-0071] Wang, W. , Kong, P. , Ma, G. , Li, L. , Zhu, J. , Xia, T. , … Wang, S. (2017). Characterization of the release and biological significance of cell‐free DNA from breast cancer cell lines. Oncotarget, 8, 43180–43191. 28574818 10.18632/oncotarget.17858 PMC5522137

[acel12734-bib-0072] Wang, Z. , & Lieberman, P. M. (2016). The crosstalk of telomere dysfunction and inflammation through cell‐free TERRA containing exosomes. RNA Biology, 13, 690–695. https://doi.org/10.1080/15476286.2016.1203503 27351774 10.1080/15476286.2016.1203503 PMC4993293

[acel12734-bib-0073] Weilner, S. , Keider, V. , Winter, M. , Harreither, E. , Salzer, B. , Weiss, F. , … Grillari, J. (2016). Vesicular Galectin‐3 levels decrease with donor age and contribute to the reduced osteo‐inductive potential of human plasma derived extracellular vesicles. Aging (Albany NY), 8, 16–33. https://doi.org/10.18632/aging.100865 26752347 10.18632/aging.100865 PMC4761711

[acel12734-bib-0074] Wells, S. I. , Aronow, B. J. , Wise, T. M. , Williams, S. S. , Couget, J. A. , & Howley, P. M. (2003). Transcriptome signature of irreversible senescence in human papillomavirus‐positive cervical cancer cells. Proceedings of the National Academy of Sciences of the United States of America, 100, 7093–7098. https://doi.org/10.1073/pnas.1232309100 12756294 10.1073/pnas.1232309100 PMC165835

[acel12734-bib-0075] Witwer K. W. , Buzás E. I. , Bemis L. T. , Bora A. , Lässer C. , Lötvall J. , … Hochberg F. (2013). Standardization of sample collection, isolation and analysis methods in extracellular vesicle research. Journal of Extracellular Vesicles 2, 20360 https://doi.org/10.3402/jev.v2i0.20360 10.3402/jev.v2i0.20360 PMC3760646 24009894

[acel12734-bib-0076] Yang, H. , Wang, H. , Ren, J. , Chen, Q. , & Chen, Z. J. (2017). cGAS is essential for cellular senescence. Proceedings of the National Academy of Sciences of the United States of America, 114, E4612–E4620. https://doi.org/10.1073/pnas.1705499114 28533362 10.1073/pnas.1705499114 PMC5468617

[acel12734-bib-0077] Yoshimoto, S. , Loo, T. M. , Atarashi, K. , Kanda, H. , Sato, S. , Oyadomari, S. , … Ohtani, N. (2013). Obesity‐induced gut microbial metabolite promotes liver cancer through senescence secretome. Nature, 499, 97–101. https://doi.org/10.1038/nature12347 23803760 10.1038/nature12347

[acel12734-bib-0078] Yu, X. , Harris, S. L. , & Levine, A. J. (2006). The regulation of exosome secretion: A novel function of the p53 protein. Cancer Research, 66, 4795–4801. https://doi.org/10.1158/0008-5472.CAN-05-4579 16651434 10.1158/0008-5472.CAN-05-4579

[acel12734-bib-0079] Yu, X. , Riley, T. , & Levine, A. J. (2009). The regulation of the endosomal compartment by p53 the tumor suppressor gene. FEBS Journal, 276, 2201–2212. https://doi.org/10.1111/j.1742-4658.2009.06949.x 19302216 10.1111/j.1742-4658.2009.06949.x

[acel12734-bib-0080] Zhang, Y. , Kim, M. S. , Jia, B. , Yan, J. , Zuniga‐Hertz, J. P. , Han, C. , & Cai, D. (2017). Hypothalamic stem cells control ageing speed partly through exosomal miRNAs. Nature, 548, 52–57. https://doi.org/10.1038/nature23282 28746310 10.1038/nature23282 PMC5999038

